# Artificial Intelligence-Assisted Identification of Genetic Factors Predisposing High-Risk Individuals to Asymptomatic Heart Failure

**DOI:** 10.3390/cells10092430

**Published:** 2021-09-15

**Authors:** Ning-I Yang, Chi-Hsiao Yeh, Tsung-Hsien Tsai, Yi-Ju Chou, Paul Wei-Che Hsu, Chun-Hsien Li, Yun-Hsuan Chan, Li-Tang Kuo, Chun-Tai Mao, Yu-Chiau Shyu, Ming-Jui Hung, Chi-Chun Lai, Huey-Kang Sytwu, Ting-Fen Tsai

**Affiliations:** 1 Division of Cardiology, Department of Internal Medicine, Chang Gung Memorial Hospital, Keelung 204, Taiwan; ningiy@gmail.com (N.-I.Y.); kuo.litang@gmail.com (L.-T.K.); 8805043@cgmh.org.tw (C.-T.M.); hmj1447@cgmh.org.tw (M.-J.H.); 2 Community Medicine Research Center, Chang Gung Memorial Hospital, Keelung 204, Taiwan; yehccl@cgmh.org.tw (C.-H.Y.); yuchiaushyu@gmail.com (Y.-C.S.); 3 College of Medicine, Chang Gung University, Taoyuan 333, Taiwan; 4 Department of Thoracic and Cardiovascular Surgery, Chang Gung Memorial Hospital, New Taipei City 333, Taiwan; 5 Advanced Tech BU, Acer Inc., New Taipei City 221, Taiwan; vincent.tsai@acer.com (T.-H.T.); Zack.Li@acer.com (C.-H.L.); Linda.Chan@acer.com (Y.-H.C.); 6 Institute of Molecular and Genomic Medicine, National Health Research Institute, Zhunan 350, Taiwan; yjchou0810@nhri.edu.tw (Y.-J.C.); paul@nhri.edu.tw (P.W.-C.H.); 7 Department of Nursing, Chang Gung University of Science and Technology, Taoyuan 333, Taiwan; 8 Department of Ophthalmology, Chang Gung Memorial Hospital, Keelung 204, Taiwan; 9 National Institute of Infectious Diseases and Vaccinology, National Health Research Institutes, Zhunan 350, Taiwan; 10 Department & Graduate Institute of Microbiology and Immunology, National Defense Medical Center, Taipei 114, Taiwan; 11 Department of Life Sciences and Institute of Genome Sciences, National Yang Ming Chiao Tung University, Taipei 112, Taiwan; 12 Aging and Health Research Center, National Yang Ming Chiao Tung University, Taipei 112, Taiwan

**Keywords:** heart failure, genetic factors, single nucleotide polymorphism, artificial intelligence

## Abstract

Heart failure (HF) is a global pandemic public health burden affecting one in five of the general population in their lifetime. For high-risk individuals, early detection and prediction of HF progression reduces hospitalizations, reduces mortality, improves the individual’s quality of life, and reduces associated medical costs. In using an artificial intelligence (AI)-assisted genome-wide association study of a single nucleotide polymorphism (SNP) database from 117 asymptomatic high-risk individuals, we identified a SNP signature composed of 13 SNPs. These were annotated and mapped into six protein-coding genes (GAD2, APP, RASGEF1C, MACROD2, DMD, and DOCK1), a pseudogene (PGAM1P5), and various non-coding RNA genes (LINC01968, LINC00687, LOC105372209, LOC101928047, LOC105372208, and LOC105371356). The SNP signature was found to have a good performance when predicting HF progression, namely with an accuracy rate of 0.857 and an area under the curve of 0.912. Intriguingly, analysis of the protein connectivity map revealed that DMD, RASGEF1C, MACROD2, DOCK1, and PGAM1P5 appear to form a protein interaction network in the heart. This suggests that, together, they may contribute to the pathogenesis of HF. Our findings demonstrate that a combination of AI-assisted identifications of SNP signatures and clinical parameters are able to effectively identify asymptomatic high-risk subjects that are predisposed to HF.

## 1. Introduction

Heart failure (HF) is an important public health problem that is associated with high morbidity, high mortality, and a burden to healthcare [1]. It is considered to be a progressive disorder and is caused by a range of different risk factors; this leads to a heterogenic pathophysiology. The symptoms of HF include effort intolerance and/or fluid retention, as well as dyspnea, fatigue, and pulmonary congestion. The American College of Cardiology and American Heart Association (ACC/AHA) have categorized HF into four stages: Stage A is defined as having risk factors for HF only; Stage B is defined as having structural heart disease without any current or prior symptoms of HF; Stage C is defined as symptomatic HF; and Stage D is HF refractory to treatment [2].

Stage B HF, which is asymptomatic, is a risk factor for mortality. Stage B HF is defined by the ACC/AHA as best characterized by an increase in the left ventricular (LV) mass (LVM), an increase in the left atrial dimensions, the presence of LV geometric patterns indicative of adverse remodeling (i.e., concentric remodeling and/or both concentric and eccentric hypertrophy), and a reduction in the LV ejection fraction (LVEF) or diastolic dysfunction. However, Stage B HF subjects are asymptomatic, thus it is highly unlikely this clinically silent population will routinely receive a detailed examination of their heart. This leads to a five-fold increase in mortality risk among such individuals, as well as the transition from Stage B HF to Stage C HF, which is associated with elevated rates of hospitalization and death [3]. The ACC/AHA guidelines have emphasized the importance of the appropriate treatment of Stage B HF subjects to prevent the development of symptomatic HF [2] and have highlighted the need for early detection strategies to identify these clinically asymptomatic Stage B HF subjects.

The risk prediction of a complex disease such as the asymptomatic Stage B HF is currently a challenge as well as is an unmet clinical need. It is well documented that the risk factors for HF include environmental factors, metabolic derangements, and genetic factors [2], and there is also an increasing appreciation that there is an underlying strong heritable component. This strengthens the importance of discovering genetic factors that contribute to the underlying mechanism in an attempt to reveal novel targets for the prevention and treatment of HF. Notably, recent studies of single nucleotide polymorphisms (SNPs) have suggested that there is an association between certain genetic factors and an increased risk of HF [4,5]. However, the role of those factors and the molecular mechanism(s) behind the pathogenesis of HF remain incompletely understood.

Artificial intelligence (AI) techniques have been applied previously to cardiovascular diseases, namely model prediction of the presence of HF, estimation of the HF subtype, and assessment of the severity of HF [6]. Currently, most studies of AI-assisted HF prediction have used clinical features and focused on the recognition of subtypes for prognosis [7,8], such as destabilizations, re-hospitalizations, and mortality [9,10]. In addition, the techniques of AI machine-learning also have great potential for delineating complex biological processes, in particular those involving interactions between the multiple genetic factors and biochemical pathways that accelerate the development of HF. Accordingly, in this study, we applied an AI-assisted methodology to identify the genetic factors in a high-risk population that are potentially associated with asymptomatic Stage B HF. This involved carrying out genome-wide SNPs screening. Furthermore, we also performed protein connectivity mapping of the genes in which the SNPs are located in order to pinpoint their potential role in the molecular pathogenesis of HF in terms of functional connectivity and protein–protein interaction networks.

## 2. Materials and Methods

### 2.1. Study Subjects

Between February 2019 and November 2019, 162 prospectively recruited participants from the Northeastern Taiwan Community Medicine Research Cohort (NTCMRC, ClinicalTrials.gov Identifier: NCT04839796) were enrolled and examined in the cardiology outpatient clinic of Chang Gung Memorial Hospital, Keelung. All subjects received a clinical examination, blood tests, electrocardiogram (ECG) and echocardiography evaluation, as well as having a complete personal and past medical history takenrecording. The 10-year and lifetime risk of atherosclerotic cardiovascular disease (ASCVD) was calculated for each subject [11]. The inclusion criteria were as follows: subjects were over 30 years old with a 10-year ASCVD risk ≥20%. The exclusion criteria were that the subject was already known to have clinical HF at Stage C or D; was suffering from atrial fibrillation (as identified by having a previous diagnosis of atrial fibrillation or paroxysmal atrial fibrillation; found to have documented atrial fibrillation on ECG or during the echocardiography exam); and/or was pregnant. Informed signed consent was obtained from all participants. This study protocol conforms to the ethical guidelines of the 1975 Declaration of Helsinki and was approved by the Institutional Review Board of Chang Gung Medical Foundation (IRB No: 201800802B0 and 202000077B0A3).

### 2.2. Clinical Assessment

At recruitment, all participants provided a detailed personal history and received a full physical examination, completed a questionnaire, and underwent various biochemical tests, including assays for n-terminal pro-brain natriuretic peptide (NT-proBNP), high sensitivity-Troponin T (hs-Tnt), and high sensitivity c-reactive protein (hs-CRP), as well as a two-dimensional (2D) echocardiography examination. Blood pressure was measured using the average of two seated measurements. Heart rate was measured via a resting 12-lead ECG. Body mass index was calculated as weight divided by height^2^ and expressed as kg/m^2^. Diabetes mellitus was defined as a fasting glucose of ≥126 mg/dL, a random glucose of ≥200 mg/dL, or the use of hypoglycemic medications. Previous history of coronary heart disease was used to identify if subjects had angina pectoris with a positive exercise test result; a history of myocardial infarction; angiographic evidence of significant (>75%) coronary artery stenosis after intra-coronary nitroglycerine 50–200 μg administration; a history of percutaneous coronary revascularization; or coronary artery bypass grafting. Current smoking status was defined as having smoked more than 100 cigarettes in their lifetime and having smoked within 1 month before enrollment.

### 2.3. Biochemical Analysis

Blood specimens were collected in citrate-treated tubes at recruitment. After centrifuging for at least 15 min, the plasma component was frozen and shipped on dry ice to the core laboratory center of our hospital, at which the samples were stored at −80 °C for the subsequent measurement of cytokines and inflammatory markers. Plasma hs-CRP was measured in duplicates by an enzyme-linked immunosorbent assay on the basis of purified protein and polyclonal anti-C-reactive protein antibodies (IMMULITE hs-CRP, Diagnostic Products Corporation, Los Angeles, CA, USA). The lower limit of this assay was 0.10 mg/L and the coefficient of variation was ≤5% at the 0.20 mg/L C-reactive protein level. The plasma concentrations of hs-Tnt and NT-proBNP were measured using appropriate sandwich enzyme-linked immunosorbent assay kits and the monoclonal antibody targeting the relevant cytokine (R&D Systems, Inc., Minneapolis, MN, USA). Other related biomarkers, namely leptin and adiponectin, were measured using commercially available enzyme-linked immunosorbent assays (Boster Biological Technology, Pleasanton, CA, USA).

### 2.4. Echocardiography

Echocardiography was performed within one month of recruitment. A comprehensive transthoracic Doppler echocardiography was performed using a commercially available machine (Vivid E9 system, General Electrics, Boston, MA, USA) with a M5S probe. LV end-diastolic and end-systolic volume were measured from the apical two-chamber and four-chamber view, and the LVEF was calculated using the modified biplane Simpson’s rule. The LVM index was measured according to the American Society of Echocardiography formula. Conventional Doppler parameters were measured according to a standardized examination procedure and these were early (E) and late diastolic transmitral flow velocity; deceleration time of E; average of the septal annular mitral early diastolic, late diastolic, and systolic tissue velocities (E’); and the ratio of E/E’. The pulmonary artery systolic pressure was calculated using the modified Bernoulli equation from the tricuspid regurgitation peak jet velocity and estimated right atrial pressure (from the respiratory variation of the inferior vena cava diameter). Two-dimensional strain analysis was performed using custom 2D strain-imaging software (EchoPac, GE Ultrasound, Boston, MA, USA). The endocardial borders were traced from the end-systolic frame of the 2D images. Interactive software then automatically tracked myocardial motion and divided each image into six segments. Numerical and graphical displays of the deformation parameters (reflecting the average value for the tracking of all acoustic markers in each segment) were then generated for all six segments from each view. Longitudinal peak systolic strain was acquired for the apical two-chamber, three chamber, and four-chamber views. If further shortening occurred after the end of the systole, this was measured as the peak strain. Global longitudinal strain was calculated as the average longitudinal strain of the segments of two-chamber, three-chamber, and four-chamber views. All examinations of echocardiography were performed and analyzed by one experienced physician (Dr. Ning-I Yang) of excellent reproducibility [12] who was blinded to the subject data.

### 2.5. Definition of Stages A and Stage B Heart Failure

HF preclinical stages were assessed based on the clinical history and echocardiographic data. Stage A HF was defined as the presence of risk factors such as arterial hypertension; type 2 diabetes; obesity; metabolic syndrome; a documented clinical history of atherosclerotic disease or the use of cardiotoxins when there was no evidence of structural heart disease; or the signs/symptoms of HF. Stage B HF was defined as the presence of structural heart disease or the detection of diastolic dysfunction on the echocardiographic examination. The latter subject needed to fulfill one of the following criteria: (1) LV hypertrophy as defined by an LV mass index of >95 g/m^2^ in women or of >115 g/m^2^ in men; (2) LV dilatation as defined by an LV end diastolic volume index of >95 mL/m^2^; (3) concentric remodeling defined by a relative wall thickness of >0.42; (4) asymptomatic LV dysfunction, including an LVEF of < 50% and/or diastolic dysfunction (≥grade II) without clinical signs and/or symptoms of HF; and (5) more than mild mitral or aortic regurgitation.

### 2.6. DNA Extraction of White Blood Cells

Peripheral venous blood was collected from the subjects and processed on the same day. Each blood sample was centrifuged at 3000 rpm for 10 min at 4 °C to separate the serum from cells. Genomic DNA was then isolated from peripheral white blood cells using the phenol/chloroform DNA extraction method after lysis of red blood cells. Finally, precipitation and washing using 95% isopropanol followed by 80% alcohol were used to obtain the total genomic DNA.

### 2.7. Whole-Genome SNP Analysis

To identify single nucleotide polymorphisms (SNPs), we genotyped the genomic DNA using Axiom^TM^ Genome-Wide TWB 2.0 array plates, which included 686,463 SNPs. Genotyping analyses were performed on 117 high-risk (ASCVD risk ≥ 20%) subjects, including 83 Stage A and 34 Stage B asymptomatic HF subjects. The stages of HF were defined by the echocardiographic examination results. Among the SNPs identified, those with a minor allele frequency rate of 0 or those SNPs with a missing rate of more than 10% were excluded from further analysis. In total, 392,885 SNPs were available for further analyses.

### 2.8. AI-Assisted Discovery of Candidate SNPs

For the model training and testing, all machine-learning analyses were performed using R Version 3.5.3 (using the random Forest, e1071, glmnet, rpart, caret, and cvAUC packages). We used three supervised algorithms to select important features, namely the random forest (RF), support vector machine (SVM), and least absolute shrinkage and selection operator (LASSO) methods, with the input dataset having a train-to-validation split ratio of 80:20. The SNPs were ranked by the summation of the selected counts using 100-time bootstrapped random samples and the three machine-learning methods. The minimum features needed were the highest performance for the area under the curve (AUC) and the accuracy rate; these were calculated for the four different machine-learning models (RF, SVM, LASSO, and the decision tree).

### 2.9. Protein Interaction Network

The locations of AI-identified SNP signatures were mapped to their relevant genes. To explore the regulatory mechanisms and potential pathways that the genes may be involved in, the six protein-coding genes from the twelve signature genes were subjected to protein–protein interaction analyses using the BioGRID database [13]. The results are displayed as a graphical network using the open-source software Cytoscape [14].

### 2.10. Statistics

Two independent sample *t*-tests were used to compare differences between the continuous variables derived from the groups. The results are presented as means ± standard deviations (SD). The χ^2^ test was used to examine the distribution of the categorical variables and results are expressed as frequencies and percentages between the groups. A multiple logistic regression model was used to determine the strength of association between the selected parameters and the presence of Stage B HF. The statistical software used for this study was SPSS 25.0 (IBM Corporation, Armonk, NY, USA).

## 3. Results

### 3.1. Clinical, Biochemical, and Echocardiography Data

**Figure 1:**
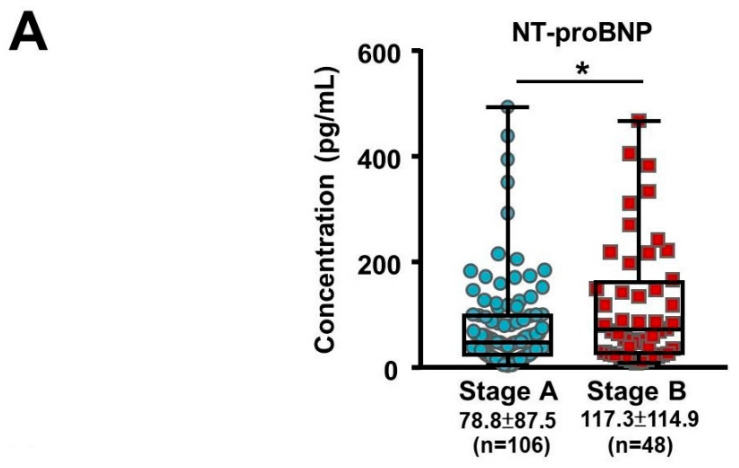

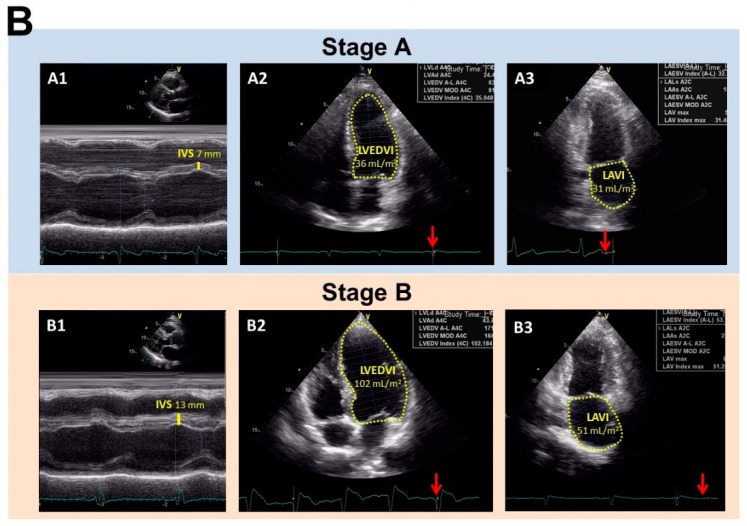
Representative echocardiographic images and the NT-proBNP levels of subjects in Stage A and B heart failure (HF). (**A**) The NT-proBNP levels of the subjects in Stage A and B HF. * indicated *p* < 0.05 by independent *t*-test. (**B**) Upper panel: a 68-year-old male subject with Stage A HF (body surface area (BSA) of 1.8 m^2^). His echocardiographic parameters, including interventricular septum thickness (IVS; A1, 7 mm, normal range 6–10 mm), left ventricle end diastolic volume index (LVEDVI; A2, 36 mL/m^2^; normal range < 95 mL/m^2^), and left atrium volume index (LAVI; A3, 31 ml/m^2^; normal range < 34 mL/m^2^) were within normal ranges. Bottom panel: a 70-year-old male subject with asymptomatic Stage B HF (BSA 1.9 m^2^). The echocardiographic examination results demonstrated myocardial remodeling, including thickened IVS (B1, 13 mm) and enlarged LVEDI (B2, 102 mL/m^2^) and LAVI (B3, 51 mL/m^2^). The red arrow indicates that the echocardiographic pictures were taken at the end-diastolic phase in A2 and B2, and at the end-systolic phase in A3 and B3.

A total of 162 subjects were recruited into the study between February 2019 and November 2019. The demographic and clinical baseline characteristics of the subjects are shown in Table 1. Of these, 113 subjects (70%) had Stage A HF and 49 subjects (30%) had Stage B (asymptomatic) HF. Both groups had a male predominance and the ASCVD risk scores in both groups were in the high category (>20%). The risk factors for HF included coronary artery disease as well as hypertension and diabetes, and these were similar for both groups. Biochemical analysis showed that the Stage B HF group had higher levels of NT-proBNP (Figure 1A, 117.35 ± 114.93 vs. 78.30 ± 87.55, *p* = 0.040) and adiponectin (13.13 ± 10.32 vs. 8.74 ± 8.86, *p* = 0.011). Our results demonstrated that elevated serum levels of NT-proBNP and adiponectin correlated with the progression of HF in asymptomatic patients. Despite the beneficial effects of adiponectin on cardiometabolic traits, increased adiponectin has also been found during HF progression, the so-called adiponectin paradox [15]. Echocardiography analysis showed that both groups had adequate LV systolic function and right ventricle systolic function, as shown by the LVEF, global longitudinal strain analysis, and tricuspid annular plane systolic excursion parameters. Since Stage B HF is defined as having LV hypertrophy, dilatation, increased relative wall thickness, and/or LVdysfunction, the corresponding echocardiographic parameters were greater in the Stage B group as would be expected (Table 2; Figure 1B). Following multiple logistic regression analyses, it was found that an elevated NT-proBNP level was associated with a prediction of Stage B HF (odds ratio: 1.005, 95% confidence interval 1.000–1.010, *p* = 0.032; Table 3).

### 3.2. AI-Assisted Identification of SNP Signature: Model Selection, Performance, and Validation

**Figure 2:**
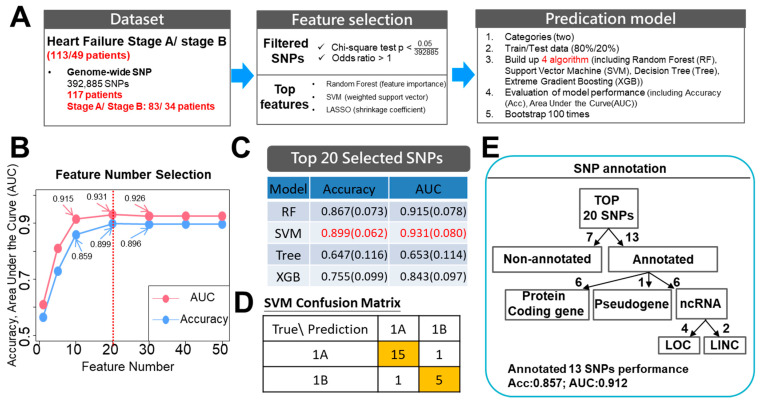
Prediction models built based on the clinical features and genome-wide SNPs were used to classify Stage A and Stage B using machine-learning methods. (**A**) Machine-learning workflow to select reliable and predictable features to build prediction models for the group classification. (**B**) The accuracy and AUC of the SVM model with different feature selection numbers. (**C**) Classification performance of the four machine-learning algorithms with selected feature subsets from genome-wide SNPs. (**D**) The SVM confusion matrix used for prediction accuracy. (**E**) Classification of selected SNPs.

Genome-wide association studies (GWAS) have been able to identify thousands of SNPs that are linked to complex human diseases. To categorize loci associated with asymptomatic HF, namely Stage B, we combined AI-assisted analysis with GWAS using the data from the 117 high-risk (ASCVD risk ≥ 20%) subjects. This study comprised three parts, which were as follows: (1) the performance of GWAS on 83 subjects with Stage A HF and 34 subjects with asymptomatic Stage B HF using the Axiom Genome-Wide TWB 2.0 Array; (2) feature selection; and (3) model derivation and validation. During the feature selection process, only SNPs with adjusted *p*-values < 1 × 10^−7^ (0.05/392885) and an odds ratio of > 1 in 392,885 SNPs, whose *p*-values were calculated by χ^2^ test and adjusted with Bonferroni correction, were used for the correlation analysis of the HF stage. Multiple feature importance methods within the various different machine-learning algorithms consisted of feature importance in the random forest (RF) approach, weighted support vector in the support vector machine (SVM) approach, and a shrinkage coefficient >0 in the least absolute shrinkage and selection operator (LASSO) approach; these were integrated into the ranking features importance. For model derivation and validation, subjects were randomly assigned into either a training (80%) or a validation (20%) set after selection of the significant SNPs by these three machine-learning (RF, SVM, and LASSO) methods. The above process was repeated 100 times using different combinations of subject to form the training and validation sets. The SNPs were ranked using their summarized counts from the 100-times random samples obtained from the three machine-learning methods (Figure 2A). Although we didn’t have a large sample size, we used three screening processes to obtain reliable results. Firstly, the adjusted *p*-value (Bonferroni correction) was used to identify the SNPs with strong evidence. Secondly, the positive odds ratio was used to provide reasonable explanations on the risk alleles of the disease. Thirdly, the bootstrap (resampling technique) method with multiple replication times was used to approximate the true population distribution. In addition, different variable selection methods, including machine-learning and statistical methods, were used to rank the importance of SNPs. Notably, our data revealed that the top-ranked SNPs were frequently picked up by the machine using the variable selection and bootstrapped random sample (reproducibility) methods. This means that, although the training and validation samples were different in every bootstrap replication, the top-ranked SNPs consistently showed their importance in distinguishing between Stage A and B patients. Of the four machine-learning models tested (RF, SVM, LASSO, and the decision tree), the SVM model gave the best performance in terms of AUC and accuracy rate. The top 20 SNPs selected by AI had the best prediction performance and the accuracy rate was 0.899 while the AUC was 0.931 when differentiating between Stage A and Stage B (Table 4; Figure 2B–D). Among the top 20 SNPs, 13 SNPs had previously been annotated and mapped either to protein-coding genes, pseudogenes, or non-coding RNA (ncRNA) genes. Furthermore, using these annotated 13 SNPs, the prediction still performed well with an accuracy rate of 0.857 and AUC of 0.912 when differentiating between Stage A and B subjects (Figure 2E).

### 3.3. The AI-Selected SNP Signature Identifies Asymptomatic High-Risk Subjects That Are Predisposed towards Progression to Heart Failure

**Figure 3:**
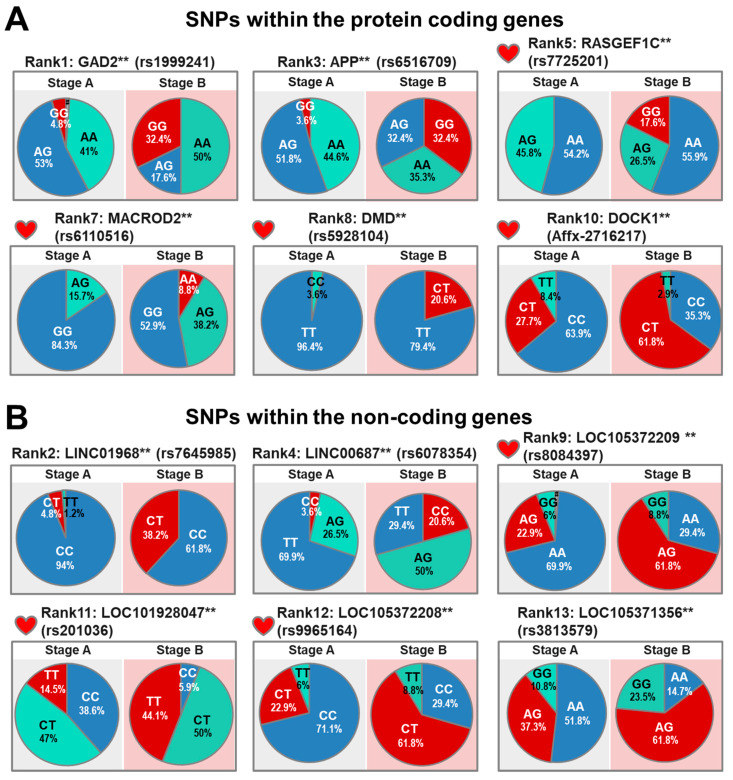
Pie charts indicating the genotype frequencies of the SNPs identified by AI-assisted analysis using the SNP datasets obtained from Stage A and B subjects. (**A**) Pie charts representing the AI-assisted SNPs within the protein-coding genes, excluding the pseudogene (PGAM1P5). Abbreviations include GAD2: glutamate decarboxylase 2; APP: amyloid beta precursor protein; RASGEF1C: RasGEF domain family member 1C; MACROD2: mono-ADP ribosylhydrolase 2; DMD: dystrophin; and DOCK1: dedicator of cytokinesis 1. (**B**) Pie charts representing the AI-assisted SNPs within the non-coding genes. rs8084397 is the SNP of LOC105372209 and LOC105372210, and rs201036 is the SNP of LOC101928047 and LOC101928004. # indicates that the signaling by the SNP array was lower than the calling rate. ** *p* < 0.005 by Chi-square test.

The haplotypes distribution, expression patterns, and functions of the genes containing the 13 SNPs that were identified using the Stage A and B subjects are summarized in Table 5. These thirteen genes include six protein-coding genes, one pseudogene, and six ncRNA genes. The SNPs in the five protein-coding genes (GAD2, APP, RASGEF1C, MACROD2, and DMD) and one pseudogene (PGAM1P5) are intron variants. The SNP located in the protein-coding gene DOCK1 is an upstream variant. When the protein-coding genes were examined, we were able to make the following observations (Figure 3A): (1) in the GAD2, APP, and RASGEF1C genes, the incidences of the G/G genotypes were significantly increased in the Stage B subjects (*p* < 0.001); (2) in the MACROD2 gene, the incidence of the A/A genotype was significantly increased in the Stage B subjects (*p* < 0.001); and (3) in the DMD and DOCK1 genes, the incidences of the C/T genotypes were significantly increased in the Stage B subjects (*p* < 0.001). Notably, expression of these four protein-coding genes, namely RASGEF1C, MACROD2, DMD and DOCK1, as well as the pseudogene PGAM1P5, can be detected in the heart [16], suggesting that these candidate genes might play a role in the pathogenesis of HF.

When the non-coding RNA (ncRNA) genes were examined, two SNPs were intron variants of long intergenic ncRNA genes (LINC01968 and LINC00687), while the remain-ing four SNPs were intron variants of ncRNA genes that have uncharacterized functions (LOC105372209, LOC101928047, LOC105372208, and LOC105371356). When the ncRNA genes were examined, we were able to make the following observations (Figure 3B): (1) in LINC01968 and LOC105372208, the C/T genotypes are significantly increased in Stage B subjects (*p* < 0.001); (2) in LINC00687, the incidence of the C/C genotype is significantly increased in Stage B subjects (*p* < 0.001); (3) in LOC105372209, LOC105372210, and LOC105371356, the A/G genotypes are significantly increased in Stage B subjects (*p* < 0.001); and (4) in the LOC101928047 and LOC101928004, the incidences of the T/T geno-types are significantly increased in Stage B subjects (*p* < 0.001). It should also be noted that the expression of three of these ncRNA, namely LOC105372209, LOC101928047, and LOC105372208, are detectable in the heart [17], which suggests that they might also be involved in HF progression.

### 3.4. The Protein Interaction Network of the Genes for which the AI-Assistance Identified where the SNPs Are Located

**Figure 4:**
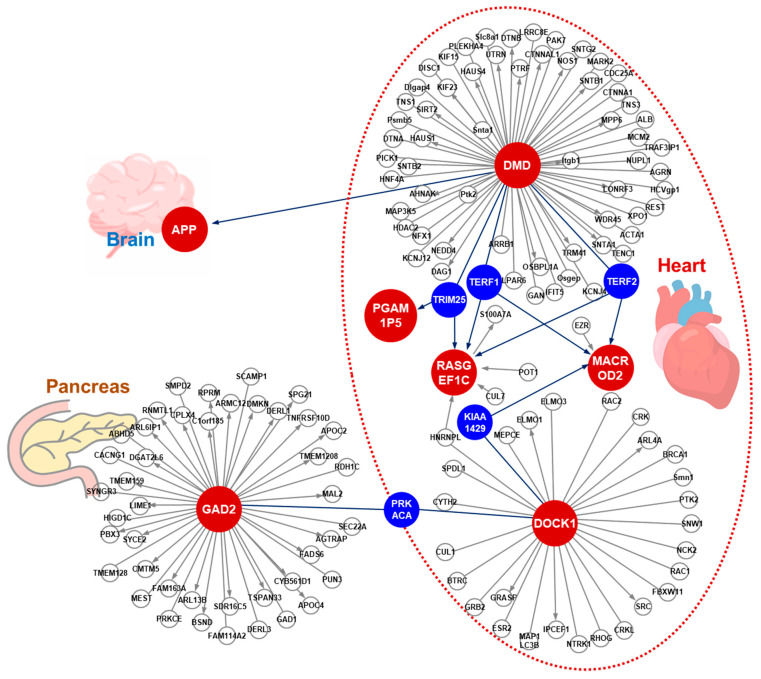
Protein–protein interaction network of the genes containing the AI-assisted identification of SNPs. The protein–protein interaction network was established using the protein-coding genes that contain a SNP. The red dots indicate the genes containing the AI-assisted SNPs and the blue dots indicate the proteins that are connected to the proteins containing SNPs within the network. PGAM1P5 is annotated as a pseudogene. Abbreviations include VIRMA: Vir-like M6A Methyltransferase-associated; PRKACA: protein kinase CAMP-activated catalytic subunit alpha; TERF1: telomeric repeat binding factor 1; TERF2: telomeric repeat binding factor 2; and TRIM25: tripartite motif containing 25.

To explore the potential role in the pathogenesis of the high-risk Stage B subjects of the identified protein-coding genes where the SNPs are located, we conducted a protein–protein interaction network analysis in order to identify any potential pathways that might affect cardiac function. Interestingly, five hub proteins, namely TERF1, TERF2, TRIM25, KIAA1429, and PRKACA, were identified in the protein–protein interaction network (marked in blue). This indicates that these proteins are likely to serve as hubs to connect DMD, RASGEF1C, MACROD2, DOCK1, and PGAM1P5 in the heart (Figure 4). In addition, within the protein interaction network, DMD seems to connect with APP, which is expressed in the brain, whereas DOCK1 seems to connect with GAD2, which is expressed in the pancreas (Figure 4). Currently, we cannot rule out the possibility that these two interactions might occur in vivo in cardiomyocytes via a novel mechanism that involves the transportation of the proteins or peptides outside the heart. For example, Aβ, the product of APP, might be released from the brain and delivered to the heart via the circulation where it can interact with DMD, thereby affecting cardiac function.

## 4. Discussion

The need to halt the progression of asymptomatic pre-clinical HF cannot be over emphasized and thus there is an urgent need for new diagnostic and management tools. The early identification of Stage B HF subjects can be challenging, despite the fact that there is a clear association between traditional risk factors and the development of HF [18]. Notwithstanding the above, the majority of individuals with hypertension and prior myocardial infarction do not eventually develop new-onset HF, a concept that is often referred to as the “prevention paradox”. In this study, we have shown that AI-assisted machine-learning is able to identify SNPs that are potentially associated with the risk of Stage B preclinical HF in high-risk individuals.

### 4.1. The Genomics of Heart Failure

The presence of heritable, polygenic components related to symptomatic and asymptomatic cardiovascular disease have been long recognized [19]. Many loci are associated with cardiovascular risk factors and diseases, and these have provided insights into the possible mechanisms that underlie the disease. However, there remains a great need for efforts to translate these genomic mechanisms into clinical practice. The application of GWAS has made the identification of loci possible, which is possibly related to the occurrence of HF and the mortality associated with HF. However, due to the heterogeneous pattern of HF, very few GWAS studies have been able to be replicated. Based on the results of GWAS studies, most cardiovascular diseases seem to be influenced by a large number of loci and these variants themselves are seldom the causal variants of the disease. Interestingly, the aggregation of these minor loci account for 10% to 36% of the inherited variation in hyperlipidemia [20], type 2 diabetes [21], myocardial infarction [22], and HF [23,24]. In this study, we hypothesized that genetic factors have an important role in the progression of asymptomatic HF in high-risk subjects. Using a traditional approach, our study subjects were sieved and we confirmed that the high-risk subjects were without any identifiable clinical manifestations of HF. The subjects included in this study were homogenous without other obvious cardiac diseases. In previous studies, the lack of homogeneity has been a potential bias. Our results provide a possible precision medicine approach to the early identification of individuals who are asymptomatic but at high risk of HF. Current guidelines are lacking on how to precisely predict the progression to HF and there is a lack of specific preventive measures or treatments that can used to help these asymptomatic high-risk patients.

### 4.2. Integrate Artificial Intelligence into the Traditional Prediction Model

In the era of precision medicine, AI or machine-learning algorithms have a number of advantages over the traditional regression model approach [25,26]. Currently, machine-learning or AI is utilized for predicting the prognosis for HF and this has used large or multifaceted datasets such as electronic health records [27] or multi-omics data [28]. However, these datasets are biased due to heterogeneous causes of HF. These prediction models created a “black-box” algorithm that shows only limited improvement over traditional logistic regression prediction models [29]. The application of AI (machine-learning) needs to be incorporated into the traditional approach rather than there being a case of mutual exclusion. In this study, we screened high-risk asymptomatic subjects using a traditional scoring system and this was then followed by AI-assisted prediction using whole genome SNPs. In previous studies, the AUCs of the prognosis prediction, generated by their identified SNPs, combined with traditional risk factors ranged from 0.56 to 0.77 [30,31,32]. Our approach using traditional risk factors, AI-assisted analysis, and a combination of 13 novel SNPs gave an AUC of 0.91. Our findings demonstrate that using an integrated application of traditional and AI-assisted approaches dramatically improves HF prediction.

### 4.3. Individual SNPs and Heart Failure Progression

Although prior studies using subjects with European ancestry have identified associations between candidate SNPs in the introns of PITX2, ABO, ACTN2, MYOZ1, SYNPO2L, BAG3, and CDKN1A [23,33], such an analysis of a combined database constructed from a number of different cohorts results in an increase in the heterogeneity of the etiology and clinical manifestation of HF; this then leads to a reduction in the statistical power [33]. Other studies focusing on a Han population found an association between a prognosis of heterogeneous cardiomyopathy with SNPs associated with LGALS3 [34], AGCT, SLC25A13, HRG, APOB, SOD3, SYNM, and TLN2 [30]. In the present study, three organ-specific clusters of SNPs were identified (Figure 4). Specifically, APP and GAD2 are mainly expressed in the brain and pancreas, respectively, while the other SNPs, namely DMD, MACROD2, PGAM1P5, RASGEF1C, and DOCK1, are mainly expressed in the heart. The GAD2 polymorphism has been reported to be associated with eating behaviors among women [35] and the risk of obesity [36,37]. Obesity is a well-known risk factor for a HF progression. Another gene associated with obesity found in our signature is the APP gene, which is upregulated in mitochondria and regulates mitochondrial function [38]. Another gene, MACROD2, which is one of three mono ADP-ribosylases in humans, has been reported to act as a transcriptional regulator of adipogenesis and obesity in a Han population [39]. The DOCK1 gene, an atypical Rac activator, has been associated with obesity in a Yup’ik population [40] and is required for cardiovascular development [41]. Mutation of DMD results in muscular dystrophy that can be complicated by the presence of HF and irregular heart rhythms [42]. Finally, there is increasing evidence that lncRNAs are able to affect the expression of protein-coding genes by competitively binding to shared miRNAs, which then reduces the degradation of protein-coding genes. Several studies have found that lncRNA-associated competing endogenous RNA cross-talks with cardiovascular disease pathogenetic processes [43,44].

### 4.4. Natriuretic Peptides and the Brain

Cardiovascular disorders share many risk factors with Alzheimer’s disease and other memory disorders. NT-proBNP has been found to be an independent risk marker for the incident of dementia and Alzheimer’s disease [45], with higher levels of NT-proBNP being significantly associated with a smaller total grey matter volume [46]. One possible explanation for the relationship between NT-proBNP and dementia may be that individuals with an elevated level of NT-proBNP are more likely to suffer from clinically identified and silent brain ischemic events. It is interesting to note that changes in NT-proBNP are still associated with dementia even after adjusting for CVD risk factors and stroke [47]. Another plausible explanation is that NT-proBNP has a role as a marker of myocardial stress, whereby it reflects the mechanisms leading to progressive subclinical cardiac dysfunction with concomitant myocardial [48] and retinal microvascular damage [49]. Finally, it should be noted that the expression of NT-proBNP is also elevated during stroke and has been found to be associated with increased mortality from stroke [50].

### 4.5. The Cardiac Natriuretic Peptide System

In our study population, we found that Stage B subjects had a higher level of NT-proBNP. It is well known that cardiac hormones and their prohormones are involved in cardiovascular hemostasis via the regulation of natriuresis, diuresis, vasodilatation, and the inhibition of the renin–angiotensin–aldosterone system (RAAS). BNP is a natriuretic hormone that was initially identified in the brain but is released mainly from the heart, particularly as a response when a ventricle is subject to high ventricular filling pressure [51]. Cleavage of the prohormone pro-BNP produces two forms; these are the biologically active 32 amino acid BNP and the biologically inactive 76 amino acid NT-proBNP. These natriuretic peptides play an important role in the diagnosis of patients who are suspected to have HF [52]. It has been shown previously that when an individual is at risk of HF, BNP-based screening and collaborative care is able to reduce the combined rates of LV systolic-diastolic dysfunction and clinical HF [53]. In addition, NT-proBNP-guided RAAS antagonists and beta-blocker therapy in diabetic subjects have been shown to be beneficial and to help prevent cardiac events [54].

### 4.6. The Heart-to-Brain Connection

Alzheimer’s disease (AD) and HF with a preserved fraction are age-related disorders that can coexist; they also have common risk factors, a similar epidemiological stratification, and involve common triggers, including oxidative stress, inflammation, and hypoxia. An examination of elderly AD patients has identified the presence of subclinical heart disease, including LV hypertrophy, aortic valve thickening, and aortic regurgitation. The hallmark of AD is the deposition of amyloid plaques, which consist primarily of a 40–42 amino acid peptide called amyloid-β (Aβ). These peptides aggregate into fibrils that then form an ordered β-sheet structure. The amyloid precursor protein is known as the precursor protein for AD-related amyloid Aβ [55]. Aβ deposition in the walls of the cerebral blood vessels is a hallmark lesion of cerebral amyloid angiopathy. In addition, APP and amyloid beta precursor such as protein 2 (APLP2) have also been found to be expressed in cardiomyocytes when heart pathology is present [56]; thus, Aβ may play a role in cardiomyocyte degeneration during HF [57]. Inclusions in the cardiomyocytes of an aging heart are described as being basophilic degenerations of the heart; this has been found to be correlated with age, the degree of myocardial fibrosis in individuals with arterial hypertension, and the severity of cerebral amyloid angiopathy. The fragments detected as part of cardiac basophilic degeneration indicate the presence of specific inclusion body pathology that is related to amyloid precursor protein metabolism. The severity of cerebral amyloid angiopathy has been found to be related to the amyloid precursor protein-derived amyloid β-protein, which suggests a possible link between myocardial and cerebrovascular amyloid precursor protein-related lesions [58].

### 4.7. Limitations and Future Perspectives

This study has several limitations. First, this is a cross-sectional study involving a limited number of subjects from north-east Taiwan and therefore the results need to be validated using both larger scale cohorts and long-term follow ups; furthermore, the findings may not be applicable to western populations. Second, the 13 SNPs’ signatures, identified by AI-assisted whole-genome SNP analysis, are associated with HF progression in high-risk subjects. Thus, more studies are needed to clarify how these SNPs affect the functions of these genes and how any relevant changes are involved in the underlying mechanisms behind HF progression.

## 5. Conclusions

This study demonstrates the potential of employing AI machine-learning models to augment traditional methods when predicting genetic predispositions to HF in a high-risk population. Knowledge of the major SNPs associated with preclinical HF provides insights into the relationships between complex pathways and also highlights various key genes that potentially are targets for risk stratification, therapy, and drug development. Our results demonstrate that the application of traditional risk stratification, followed by AI-assisted analysis, is able to raise prediction performance when used on well-characterized, homogenous, and phenotypically identified subjects.

## Figures and Tables

**Table 1:** Baseline characteristics of both groups.

	Stage A HF ^1^ N = 113	Stage B HF N = 49	*p*-Value
Age, years	68.35 ± 6.19	69.88 ± 6.53	0.156
Male, n (%)	72 (63.7)	32 (65.3)	1.00
BMI ^2^, kg/m ^2^	26.60 ± 3.76	27.21 ± 3.80	0.342
Smoking, n (%)	22 (19.5)	12 (24.5)	0.53
ASCVD ^3^ risk score, %	24 ± 11.25	27.46 ± 11.83	0.087
Coronary artery disease, n (%)	34 (30.1)	11 (22.4)	0.35
Hypertension, n (%)	93 (82.3)	44 (89.9)	0.343
Diabetes mellitus, n (%)	40 (35.4)	16 (32.7)	0.858
Urine Alb/Cre ^4^ (mg/g)	67.21 ± 173.22	175.26 ± 379.36	0.084
NT-proBNP ^5^ (pg/mL)	78.30 ± 87.55	117.35 ± 114.93	0.040
Hs-Tnt ^6^ (ng/L)	10.37 ± 5.47	12.70 ± 9.67	0.128
HbA1c ^7^ (%)	6.44 ± 9.85	6.58 ± 1.56	0.549
Creatinine (mg/dL)	1.00 ± 0.27	1.06 ± 0.40	0.287
AST ^8^ (U/L)	24.79 ± 11.44	30.80 ± 37.52	0.277
ALT ^9^ (U/L)	29.05 ± 17.13	38.19 ± 66.93	0.351
Sodium (mEq/L)	141.41 ± 1.92	141.65 ± 2.56	0.501
Potassium (mEq/L)	4.33 ± 0.40	4.27 ± 0.41	0.393
Albumin (g/dL)	4.58 ± 0.23	4.52 ± 0.29	0.194
Total bilirubin (mg/dL)	0.68 ± 0.28	0.66 ± 0.25	0.561
Total protein (g/dL)	7.36 ± 0.40	7.31 ± 0.36	0.494
Uric acid (mg/dL)	6.03 ± 1.48	5.73 ± 1.55	0.252
HDL ^10^ (mg/dL)	51.83 ± 13.4	52.24 ± 15.28	0.863
LDL ^11^ (mg/dL)	111.71 ± 26.09	111.02 ± 26.66	0.879
Total cholesterol (mg/dL)	178.60 ± 28.71	180 ± 35.05	0.758
Insulin (μIU/mL)	14.29 ± 7.50	13.79 ± 8.67	0.715
Hs-CRP (mg/L) ^12^	2.69 ± 11.00	2.21 ± 2.30	0.772
Ferritin (ng/mL)	303.39 ± 240.80	280.78 ± 186.70	0.559
Adiponectin (μg/mL)	8.74 ± 8.86	13.13 ± 10.32	0.011
Leptin (ng/mL)	11.47 ± 11.41	10.77 ± 9.65	0.708

^1.^ HF: heart failure; ^2^ BMI: body mass index; ^3^ ASCVD: atherosclerotic cardiovascular disease; ^4^ Alb/Cre: albumin creatinine ratio; ^5^ NT-proBNP: N-terminal pro b-type natriuretic peptide; ^6^ Hs-Tnt: high-sensitivity Troponin T; ^7^ HbA1c: hemoglobin A1c; ^8^ AST: aspartate aminotransferase; ^9^ ALT: alanine aminotransferase; ^10^ HDL: high-density lipoprotein; ^11^ LDL: low-density lipoprotein; and ^12^ Hs-CRP: high-sensitivity C-reactive protein.

**Table 2:** Echocardiographic parameters of both groups.

	Stage A HF ^1^ N = 113	Stage B HF N = 49	*p*-Value
Left atrial volume index (mL/m^2^)	34.61 ± 9.62	42.92 ± 12.32	**<0.001**
Left ventricle EDVI ^2^ (mL/m^2^)	54.22 ± 12.21	62.39 ± 19.79	**0.009**
Left ventricle ESVI ^3^ (mL/m^2^)	21.10 ± 5.69	23.64 ± 9.10	0.075
Left ventricle EF ^4^, 2D (%)	63.54 ± 6.58	63.92 ± 6.18	0.736
Left ventricle mass index (g/m^2^)	75.76 ± 17.56	104.45 ± 28.73	**<0.001**
Relative wall thickness	0.28 ± 0.06	0.29 ± 0.07	0.201
Peak GLS ^5^ (%)	−17.22 ± 3.06	−18.04 ± 3.31	0.134
Mitral valve E ^6^ velocity (cm/s)	0.69 ± 0.19	0.74 ± 0.20	0.207
Mitral valve A ^7^ velocity (cm/s)	0.84 ± 0.23	0.96 ± 0.20	**0.003**
Mitral valve deceleration time (ms)	204.45 ± 64.00	201.50 ± 47.24	0.775
Mitral valve E/A ratio	1.28 ± 3.36	0.78 ± 0.21	0.306
Tissue doppler septal S’ ^8^ (cm/s)	6.28 ± 1.30	5.98 ± 1.12	0.177
Tissue doppler septal E’ ^9^ (cm/s)	5.98 ± 1.12	4.95 ± 1.37	**<0.001**
Septal E/E’	11.97 ± 3.74	15.26 ± 3.66	**<0.001**
TAPSE ^10^ (mm)	22.46 ± 3.22	27.37 ± 34.28	0.307

^1.^ HF: heart failure; ^2^ EDVI: end-diastolic volume index; ^3^ ESVI: end-systolic volume Index; ^4^ EF: ejection fraction; ^5^ GLS: global longitudinal strain; ^6^ E: early diastolic transmitral flow; ^7^ A: late diastolic transmitral flow; ^8^ E’: septal annulus mitral early diastolic tissue velocity; ^9^ S’: septal annulus mitral systolic tissue velocity; and ^10^ TAPSE: tricuspid annular plane systolic excursion.

**Table 3:** Multiple logistic regression analysis for Stage B heart failure prediction.

	Odds Ratio	95% Confidence Interval	*p* Value
Male	1.456	0.543–3.902	0.455
Hypertension	1.976	0.574–6.805	0.280
Diabetes Mellitus	0.851	0.329–2.201	0.740
Smoking	2.396	0.836–6.862	0.104
Urine Alb/Cre ^1^	1.002	1.000–1.003	0.065
NT-proBNP ^2^	1.005	1.000–1.010	0.032
Adiponectin	1.031	0.986–1.078	0.182
GLS ^3^	0.929	0.809–1.067	0.298

^1^ Alb/Cre: albumin creatinine ratio; ^2^ NT-proBNP: N-terminal pro b-type natriuretic peptide; and ^3^ GLS: global longitudinal strain.

**Table 4:** Top 20 SNPs of both groups.

SNPs	Position	Gene	Allele	Stage A HF N = 113	Stage B HF N = 49	*p*-Value
rs1999241	chr10:26277498	GAD2	A A/G A/G G/0 0	34/44/4/1	17/6/11/0	<0.001
rs7645985	chr3:194777389	LINC01968	C C/T C/T T	78/4/1	21/13/0	<0.001
rs6516709	chr21:25893395	APP	A A/G A/G G	37/43/3	12/11/11	<0.001
rs6078354	chr20:11820605	LINC00687	C C/C T/T T	3/22/58	7/17/10	<0.001
rs7725201	chr5:180102124	RASGEF1C	A A/G A/G G	45/38/0	19/9/6	<0.001
rs10859918	chr12:95652910	PGAM1P5	G G/T G/T T/0 0	45/26/11/1	8/23/3/0	<0.001
rs6110516	chr20:15097697	MACROD2	A G/G G/A A	13/70/0	13/18/3	<0.001
rs4693641	chr4:83755117	None	T T/C C/C T	83/0/0	27/1/6	<0.001
rs5928104	chrX:32899133	DMD	C C/T T/C T	3/80/0	0/27/7	<0.001
rs8084397	chr18:76251014	LOC105372209; LOC105372210	A A/G A/G G/0 0	58/19/5/1	10/21/3/0	<0.001
rs4715127	chr6:49320956	None	C C/C T/T T	6/22/55	0/22/12	<0.001
rs2496369	chr6:49156247	None	G G/G T/T T	1/25/57	4/19/11	<0.001
Affx-2716217	chr10:126685478	DOCK1	C C/T C/T T	53/23/7	12/21/1	<0.001
rs56352414	chr22:48917877	None	C C/T C/T T	50/32/1	16/10/8	<0.001
rs2806810	chr13:103997325	None	A A/C A/C C	38/42/3	9/16/9	<0.001
rs4934985	chr10:33616333	None	C C/C T/T T/0 0	5/30/48/0	12/13/8/1	<0.001
rs6912291	chr6:110065139	None	C C/C T/T T	1/34/48	6/3/25	<0.001
rs201036	chr6:6708885	LOC101928047; LOC101928004	C C/T C/T T	32/39/12	2/17/15	<0.001
rs9965164	chr18:76220796	LOC105372208	C C/T C/T T	59/19/5	10/21/3	<0.001
rs3813579	chr16:79715379	LOC105371356	A A/G A/G G	43/31/9	5/21/8	<0.001

Allele: ‘0’ is no allele appearance.

**Table 5:** Stage A vs. Stage B: feature importance.

Rank	SNPs	Type	Gene	Gene Type	Function	Expression Pattern
1	rs1999241	Intron variant	GAD2	Protein-coding	Major autoantigen in insulin-dependent diabetes	Pancreas and brain
2	rs7645985	Intron variant	LINC01968	lcRNA ^1^	N/A	Testis and placenta
3	rs6516709	Intron variant	APP	Protein-coding	Neurite growth, neuronal adhesion, and axonogenesis	Brain
4	rs6078354	Intron variant	LINC00687	lcRNA ^1^	N/A	Testis
5	rs7725201	Stop gained	RASGEF1C	Protein-coding	Guanine nucleotide exchange factor	Ubiquitous
6	rs10859918	Intron variant	PGAM1P5	Pseudo	Pseudogene	Ubiquitous
7	rs6110516	Intron variant	MACROD2	Protein-coding	Removing ADP-ribose from mono-ADP-ribosylated proteins	Ubiquitous
8	rs5928104	Intron variant	DMD	Protein-coding	The DGC ^3^ bridges the inner cytoskeleton and ECM ^4^ Involved in DMD ^5^, BMD ^6^, or cardiomyopathy	Ubiquitous
9	rs8084397	Intron variant	LOC105372209 and LOC105372210	ncRNA ^2^	N/A	Heart, testis, placenta, and brain
10	Affx-2716217	Upstream variant	DOCK1	Protein-coding	Dedicator of the cytokinesis protein Phagocytosis and cell migration	Ubiquitous
11	rs201036	Non-coding transcript; intron variant	LOC101928047 and LOC101928004	ncRNA ^2^	N/A	Heart, kidney, bone marrow, and fat
12	rs9965164	Non-coding transcript	LOC105372208	ncRNA ^2^	N/A	Heart, testis, and placenta
13	rs3813579	Intron variant	LOC105371356	ncRNA ^2^	N/A	Liver, testis, kidney, and skin

^1^ lcRNA: long intergenic non-protein-coding RNA; ^2^ ncRNA: uncharacterized model non-protein-coding RNA; ^3^ DGC: dystrophin-glycoprotein complex; ^4^ ECM: extracellular matrix; ^5^ DMD: Duchenne muscular dystrophy; and ^6^ BMD: Becker muscular dystrophy.

## Data Availability

Due to ethical restrictions, the data presented in this study are available on request from the corresponding author.
